# A Case of Acute Pulmonary Embolus after mRNA SARS-CoV-2 Immunization

**DOI:** 10.3390/vaccines9080903

**Published:** 2021-08-14

**Authors:** Nathaniel E. Wiest, Gretchen S. Johns, Eric Edwards

**Affiliations:** 1 Department of Internal Medicine, Mayo Clinic, 4500 San Pablo Rd., Jacksonville, FL 32224, USA; wiest.nathaniel@mayo.edu; 2 Division of Laboratory Medicine and Pathology, Mayo Clinic, 4500 San Pablo Rd., Jacksonville, FL 32224, USA; johns.gretchen@mayo.edu; 3 Department of Hospital Internal Medicine, Mayo Clinic, 4500 San Pablo Rd., Jacksonville, FL 32224, USA

**Keywords:** COVID-19, SARS-CoV-2, vaccination, pulmonary embolus, adverse event

## Abstract

Vaccination against severe acute respiratory syndrome coronavirus 2 (SARS-CoV-2), the causative agent of coronavirus disease 2019 (COVID-19), is a critical strategy to overcome the COVID-19 pandemic. Multiple SARS-CoV-2 vaccines have been developed in a rapid timeframe to combat the pandemic. While generally safe and effective, rare cases of venous thromboembolism (VTE) have been reported after two adenovirus-based vaccines, the AstraZeneca ChAdOx1 nCoV-19 vaccine and the Janssen Ad.26.COV2.S vaccine, as well as after the Pfizer-BioNTech BNT162b2 mRNA vaccine. Here, we present the case of a patient who developed acute pulmonary emboli (PE) shortly after his second dose of the Moderna mRNA-1273 SARS-CoV-2 vaccine. We report the results of an extensive thrombophilia workup that was normal except for the identification of positive lupus anticoagulant (LA) signals. It is our goal to contribute to the body of knowledge regarding SARS-CoV-2 vaccines and encourage vaccine adverse event reporting so that clinicians can have a full appreciation and awareness of the possible adverse events related to these critical vaccines.

## 1. Introduction

The COVID-19 pandemic has claimed nearly four million lives and disrupted societies and economies globally [1]. Vaccines against SARS-CoV-2 were developed in record time and across a variety of vaccine platforms, including adenovirus vectors, protein subunit, and newer mRNA-based platforms [2]. Recently, reports of rare venous thromboembolism (VTE) and thrombocytopenia events were described for two adenovirus-based vaccines, the AstraZeneca ChAdOx1 nCoV-19 vaccine and the Janssen Ad.26.COV2.S vaccine, leading to further regulatory and safety review [3,4,5]. Furthermore, two cases of VTE were recently described shortly after patients received the Pfizer-BioNTech BNT162b2 mRNA vaccine [6,7]. While these events are fortunately rare, VTE is a potentially life-threatening adverse event whose prompt recognition is critical. Here, we describe a case of acute PE that occurred shortly after the second dose of the Moderna mRNA-1273 SARS-CoV-2 vaccine. 

## 2. Case Report

A 66-year-old male with past medical history of hypertension, hyperlipidemia, and surgically resected renal cell carcinoma (RCC) presented to the emergency department with a chief complaint of right flank pain and right pleuritic chest pain. He was in his usual state of health until he received his second dose of the Moderna SARS-CoV-2 vaccine dose 10 days prior. 24 h after the immunization he experienced fevers, chills, and arthralgias that transitioned to progressive right-sided flank pain and pleuritic chest pain. There was no history of recent immobility or surgery, and he was exercising daily before presenting. He denied any personal or family history of VTE, PE, or known hypercoagulable state. His oncologic history was notable for clinical stage II, pathologic stage T1aNXM0 RCC status-post left partial nephrectomy two years prior with clear surgical margins and no evidence of recurrence on surveillance imaging. His age-appropriate cancer screening was up to date. A full review of systems was positive only for fatigue, pleuritic chest pain, and flank pain and was otherwise negative.

**Figure 1:**
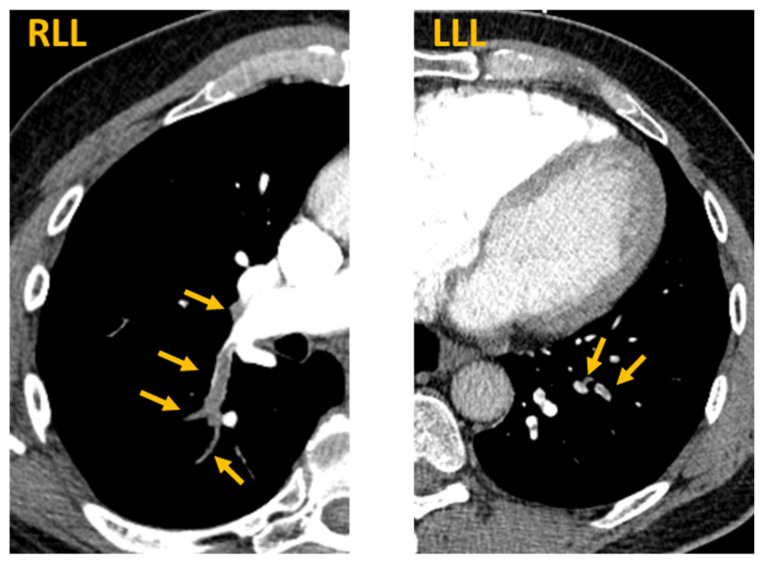
Acute pulmonary emboli after SARS-CoV-2 mRNA vaccination. CT PE protocol axial images demonstrate bilateral segmental and subsegmental pulmonary emboli. Arrows indicate contrast filling defects at locations of pulmonary emboli. RLL = right lower lobe. LLL = left lower lobe.

In the emergency department, the patient’s vital signs were normal with a temperature of 36.8 degrees Celsius, pulse rate of 65, blood pressure of 138/84 mmHg, respiratory rate of 17, and oxygen saturation of 97% on room air. Inspiratory crackles were heard at the right lung base; physical examination was otherwise unremarkable including no jugular venous distension and no extremity edema. Laboratory analysis is described in Table 1. Briefly, the patient had a normal chemistry panel and a normal complete blood count. However, he had elevated C-reactive protein and D-dimer levels. CT scan of the abdomen and pelvis without contrast demonstrated no acute intraabdominal process, including no evidence of recurrent RCC. However, PE-protocol CT angiogram of the chest demonstrated extensive multifocal pulmonary emboli involving both right and left lower lobe pulmonary arteries with evidence of right ventricular strain (Figure 1).

Following the diagnosis of PE, a 1 mg/kg subcutaneous injection of enoxaparin was immediately administered by the emergency department and labs were drawn approximately three hours later by the admitting team. A coagulation evaluation and thrombophilia workup were initiated (Table 1). The patient’s prothrombin time (PT) and international normalized ratio (INR) were mildly prolonged and corrected when mixed 50:50 with normal pooled plasma, consistent with a possible factor deficiency. However, measured factor levels were within normal limits. The patient’s prothrombin G20210A and factor V Leiden R506Q mutation tests were negative, and he demonstrated normal protein C activity, free protein S antigen, protein S activity, and antithrombin activity. A dilute PT assay was positive, and the StaClot LA assay was borderline positive, suggesting the possible presence of LA. Beta-2 glycoprotein 1 antibodies and antiphospholipid IgG antibodies were undetected; however, antiphospholipid IgM antibodies were weakly positive. SARS-CoV-2 nucleocapsid antibodies were undetected while SARS-CoV-2 spike antibodies were strongly positive, consistent with robust vaccine-induced immunity but no history of COVID-19. Antinuclear antibodies were negative and serum homocysteine levels were normal. The patient was transitioned to apixaban for anticoagulation and discharged in stable condition with hematology follow up.

## 3. Discussion

In this report, we describe the case of a 66-year-old male with no prior thromboembolic or hypercoagulable history who developed acute, bilateral pulmonary emboli promptly following his second Moderna SARS-CoV-2 immunization. One possibility is that these two events are coincidental, as there is a known 1.3–1.8 per thousand baseline annual incidence of VTE in patients age 65–69 [8]. However, the striking overlap of systemic symptoms from the mRNA immunization and the flank and pleuritic chest pain attributed to the PE are suggestive of a possible association. 

There is significant interest in the topic of VTE after COVID-19 immunization based on reports of a rare syndrome of VTE and thrombocytopenia in patients who received the adenovirus-platform AstraZeneca ChAdOx1 and Janssen Ad.26.COV2.S vaccines [3,4]. Scully et al. described 23 patients who received the AstraZeneca ChAdOx1 nCoV-19 vaccine in the United Kingdom and developed thrombosis and thrombocytopenia. Furthermore, 22 of 23 patients with this syndrome, which the authors entitled vaccine-induced immune thrombocytopenia (VITT), were found to have circulating platelet factor 4 (PF4) antibodies [4]. Greinacher et al. described 11 patients in Germany and Austria who developed VITT after the AstraZeneca ChAdOx1 nCoV-19 vaccine, and Schultz et al., described five patients in Norway who developed VITT after the AstraZeneca ChAdOx1 nCoV-19 vaccine [9,10]. All of the patients in these two reports who were tested for PF4 antibodies had positive ELISA results. Similarly, a cohort of patients who developed VTE after the Janssen Ad.26.COV2.S vaccine in the United States had a syndrome of cerebral venous thrombosis (CVT) and thrombocytopenia in 12/15 cases, though PF4 antibody levels were not reported [3]. Those authors described the syndrome as thrombosis with thrombocytopenia syndrome (TTS). PF4 antibodies are usually found in heparin-induced thrombocytopenia (HIT), a dangerous thrombotic condition that typically occurs after heparin exposure and is mediated partly by PF4 immune complexes that activate platelets and promote thrombin generation [11,12]. Thrombophilia, antinuclear antibody, and antiphospholipid antibodies were negative in the UK VITT patients, though 5/10 patients tested for LA had a positive result that was considered potentially unreliable [4]. Amongst the case series of the VITT/TTS patients, notable similarities include that most of the patients were young (<50 years old), female (61–100%), and many developed either disseminated intravascular coagulation or multiple sites of thrombosis including portal vein and CVT leading to serious illness with mortality in 20–60% of cases [3,4,9,10].

In contrast, there have been comparatively few published reports of VTE after mRNA SARS-CoV-2 immunizations. Carli et al. described a case of a 66-year-old woman who developed a right calf DVT two days after the second dose of the Pfizer-BioNTech BNT162b2 vaccine [6]. Al-Maqbali et al. described the case of a 59-year-old woman who developed DVT and PE seven days following the first dose of the Pfizer-BioNTech BNT162b2 vaccine [7]. That patient’s laboratory testing was notable for normal platelets (182 × 10^9^/L) but a positive HIT ELISA test with an optical density assay of 0.617 (normal <0.4). HIT functional confirmatory assays were not available in that report, and previous platelet levels were not reported to allow an assessment for possible decline in platelet count after immunization. Notably, that patient was hospitalized for COVID-19 pneumonia seven months prior to the immunization, and it is unclear if she had exposure to heparin products. Nonetheless, the findings of the patient described by Al-Maqbali et al. contrast with the VITT/TTS patients who experienced both severe thrombocytopenia and positive HIT ELISAs. Neither of these case reports described thrombophilia test results and both patients were treated successfully with oral anticoagulants. 

We did not test for PF4 antibodies in our patient as we had no clinical suspicion for HIT and VITT/TTS after COVID-19 immunization was not yet described. Unfortunately, no stored serum remained from our patient’s hospital encounter for us to send for PF4 antibody testing after VITT/TTS was described. Our patient’s D-dimer level was 3840 ng/mL fibrinogen equivalent units (FEU), whereas the median D-dimer level in the VITT patients was 31,301 FEU, or approximately ten times that of our patient [4,13]. This, combined with the fact that our patient had a normal platelet count, significantly reduces the likelihood that VITT/TTS was the mechanism of thrombosis in our patient, though we cannot rule out a mechanism similar to that of the Al-Maqbali et al. report with normal platelets and a positive HIT ELISA [7]. We look forward to the publication of more reports of VTE after SARS-CoV-2 immunizations so that a better assessment of potential mechanisms and comparison of patient characteristics and laboratory data can be performed.

We note that the only positive signals from the thrombophilia workup in our patient were found in the LA workup and included a positive dilute PT study, a borderline positive StaClot LA assay, and a weakly positive antiphospholipid IgM level. In the dilute PT test, patient plasma is added to two different dilutions of thromboplastin. This decreases the amount of phospholipid and enhances sensitivity to the presence of a lupus anticoagulant, which if present will prolong the patient’s PT compared to a corresponding pooled normal plasma PT [14]. Our patient had a strong positive result for the dilute PT study. In the StaClot LA test, patient plasma and normal plasma are mixed 1:1 and the aPTT is assessed both in the presence and absence of hexagonal phase phospholipid (HEX) that neutralizes LA antibodies. A positive StaClot test signifies that the HEX reagent was able to decrease the rate of clot formation for the aPTT, implying the presence of LA [15]. Our patient had a borderline positive StaClot result.

Antiphospholipid antibodies are autoantibodies that target proteins binding to phospholipids such as the cellular lipid bilayer [16]. A pathologic quantity of antiphospholipid antibodies is usually defined as >40 mean phospholipid units (MPU), whereas our patient had a weakly positive result of 27.1 MPU of antiphospholipid IgM [17]. Taken together, these studies imply the presence of LA but do not yet meet the diagnostic threshold for antiphospholipid syndrome (APS), which requires positive LA testing on two or more occasions at least 12 weeks apart [17]. Possible explanations for our patient’s LA signals include an early APS, the transient presence of a LA, or potentially false positives either due to nonspecific cross reactivity by other antibodies in the patient’s plasma or interference from the injection of enoxaparin that was given before the labs were drawn. Of note, therapeutic dose enoxaparin is not expected to affect either the dilute PT test, StaClot assay, or antiphospholipid antibody levels [18]. Repeat testing, including for PF4 antibodies, will be performed six months after PE diagnosis after the patient is off anticoagulation for two weeks to eliminate the possible interference from anticoagulants.

Interestingly, antiphospholipid antibodies have been correlated to VTE in patients with COVID-19 [19,20]. If a relationship between VTE and positive LA tests after mRNA SARS-CoV-2 immunization is reported in other patients, then this may suggest at least one similarity in mechanism of VTE between COVID-19 infection and mRNA immunization. Another possibility is that some patients with an underlying autoimmune predisposition may have flare ups after mRNA immunization, as suggested by reports of flares in conditions including familial thrombocytopenia [21], though data assessing the relationship between mRNA SARS-CoV-2 immunization and autoimmune conditions is currently lacking.

It is very important to emphasize here that the risk of VTE after SARS-CoV-2 immunization is significantly lower than the risk of VTE during COVID-19 infection, which is approximately 21% per infection in a recent meta-analysis [22]. In fact, VTE was not reported as an adverse event during Phase III studies of the Pfizer-BioNTech and Moderna SARS-CoV-2 mRNA vaccines, illustrating the rarity of these events [23,24]. While large scale epidemiologic data comparing the risk of VTE after COVID-19 infection versus mRNA immunization is not widely available yet, one pre-publication retrospective cohort study in the United Kingdom reports that the incidence of CVT was 39.0 per million people over a 2-week period after COVID-19 diagnosis versus 4.1 per million people over a 2-week period after receiving either the Pfizer-BioNTech BNT162b2 or Moderna mRNA-1273 vaccines [25]. Given that the background risk of CVT was 0.41 per million per 2-week period in this study, these data potentially support the hypothesis that there may be an increased risk of VTE after COVID-19 mRNA immunization that is significantly less than the risk incurred by natural COVID-19 infection. Additionally, data from the WHO VigiBase database indicate a rate of 0.21 cases of thrombotic events per 1 million person vaccinated-days in patients who received either the Pfizer, Moderna, or AstraZeneca vaccines [26]. Interestingly, the proportion of arterial thrombotic events was higher in the Pfizer and Moderna (mRNA) vaccine groups than the AstraZeneca (adenovirus) group, supporting the hypothesis that the mechanism of vaccine induced thrombosis is likely different between adenovirus and mRNA platform vaccines. Of note, these data were not compared to matched unvaccinated controls and as such cannot be used to compare to a background rate of venous and arterial thrombosis [26]. We eagerly await the final published version of the UK report as well as the publication of epidemiologic data from other countries including the United States. As more clinicians report adverse events and laboratory data from VTE events after COVID immunization, a better understanding of the magnitude and possible mechanisms of these adverse events will be obtained.

## 4. Conclusions

In summary, we encourage clinicians to remain vigilant for adverse events after SARS-CoV-2 immunization and to report these events to the Vaccine Adverse Event Reporting System (VAERS) in the United States or the equivalent system in other countries [27]. Given the rarity of VTE after SARS-CoV-2 mRNA immunization, we wholeheartedly endorse continued immunization in line with national and international guidelines as well as full reporting of possible vaccine related adverse events to build a better understanding of the adverse effect profiles of these critically important vaccines.

## Figures and Tables

**Table 1:** Laboratory studies. Indicated tests with patient’s laboratory values, reference ranges, and units are provided. Abnormal values highlighted in orange. WBC = white blood cell. BUN = blood urea nitrogen. GFR = glomerular filtration rate. BSA = body surface area. ESR = eosinophil sedimentation rate. PT = prothrombin time. INR = international normalize ratio. APTT = activated partial thromboplastin time. DRVVT = dilute Russell viper venom time. MPL = IgM phospholipid units. GPL = IgG phospholipid units. Ab = antibody.

Laboratory Studies			Coagulation Studies			Thrombophilia Studies		
Test	Value	Reference	Test	Value	Reference	Test	Value	Reference
**Hemoglobin**	13.8 g/dL	13.2–16.6 g/dL	**PT**	14.0 s	9.4–12.5 s	**Prothrombin G20210A mutation**	Negative	Negative
**Platelet Count**	176 × 10^9^/L	135–317 × 10^9^/L	**INR**	1.2	0.9–1.1	**Factor V leiden (R506Q) mutation**	Negative	Negative
**WBC count**	9.2 × 10^9^/L	3.4–9.6 × 10^9^/L	**APTT**	35 s	25–37 s	**Antithrombin activity**	81%	80–130%
**Sodium**	141 mmol/L	135–145 mmol/L	**Factor II Assay**	120%	75–145%	**Protein C activity**	102%	70–150%
**Potassium**	4.2mmol/L	3.6–5.2 mmol/L	**Factor V Assay**	119%	70–165%	**Protein S antigen, Free**	105%	65–160%
**Chloride**	104 mmol/L	98–107 mmol/L	**Factor VII Assay**	76%	65–180%	**Protein S activity**	137%	65–160%
**BUN**	17 mg/dL	8–24 mg/dL	**Factor X Assay**	105%	70–150%	**PT dilution 1:2**	12.4s	9.4–12.5 s
**Creatinine**	1.14 mg/dL	0.74–1.35 mg/dL	**Thrombin Time**	20.9s	15.8–24.9 s	**Dilute PT 1:50 ratio**	1.7	<1.1
**Glucose**	104 mg/dL	70–140 mg/dL				**Dilute PT 1:500 ratio**	1.7	<1.1
**Estimated GFR.**	77 mL/min/BSA	>60 mL/min/BSA				**DRVVT ratio**	1.1	0–1.1
**C-reactive protein**	38.2 mg/L	<8.0 mg/L				**StaClot LA**	10.7 s	<8 s
**ESR**	14 mm/1 h	0–22 mm/1 h				**Homocysteine**	14.4 nmol/L	7.1–16.3 nmol/L
**D-dimer**	3840 ng/mL FEU	<500 ng/mL FEU				**Antinuclear Ab**	0.1 U	<1.0 U
**SARS-CoV-2 RNA, Nasal Swab**	Negative	Negative				**Beta 2 Glycoprotein 1 Ab IgM**	<9.4 U/mL	<15.0 U/mL
**SARS-CoV-2 Nucleocapsid Total Ab**	Negative	Negative				**Beta 2 Glycoprotein 1 Ab IgG**	<9.4 U/mL	<15.0 U/mL
**SARS-CoV-2 Spike Ab**	>2500 U/mL	<0.8 U/mL				**Phospholipid Ab IgM**	27.1 MPL	<15.0 MPL
						**Phospholipid Ab IgG**	<9.4 GPL	<15.0 GPL
